# Molecular and Epidemiological Investigation of *Cryptosporidium* Infection in Goat Population from Bouira Province, Algeria

**DOI:** 10.3390/pathogens14060597

**Published:** 2025-06-18

**Authors:** Samia Bedjaoui, Djamel Baroudi, Karim Tarik Adjou, Bernard Davoust, Younes Laidoudi

**Affiliations:** 1 UMR D257 RITMES (Aix-Marseille University, Public Assistance-Marseille Hospitals, French Military Health Service), 13005 Marseille, France; samia.bedjaoui@hotmail.com (S.B.); bernard.davoust@gmail.com (B.D.); 2 University Hospital Institute Mediterranean Infection, 13005 Marseille, France; 3 Food Hygiene, Food Safety and Quality Assurance Laboratory (HASAQ), National Veterinary Higher School, Algiers 16000, Algeria; d.baroudi@ensv.dz; 4 UMR BIPAR (Anses, INRAE, Alfort National Veterinary School), 94700 Maisons-Alfort, France; karim.adjou@vet-alfort.fr; 5 PADESCA Laboratory, Veterinary Science Institute, University Constantine 1, El Khroub 25100, Algeria

**Keywords:** *Cryptosporidium xiaoi*, cryptosporidiosis, phylogeny, climate, goats, Bouira province, Algeria

## Abstract

Cryptosporidiosis is a gastrointestinal disease affecting terrestrial and aquatic vertebrates worldwide. This study investigated molecularly and microscopically the prevalence and the diversity of *Cryptosporidium* spp. in goats across the Bouira communes, Algeria. A total of 559 fecal samples were collected from 70 farms, representing 16.6% of the regional goat population. Samples were analyzed using microscopy (modified Ziehl-Neelsen staining) and molecular methods (i.e., qPCR and nested PCR targeting the 18S rRNA gene, followed by sequencing). Microscopy detected *Cryptosporidium* in 6.1% of samples, while qPCR revealed a significantly higher prevalence of 13.6% (*p* < 0.00001), confirming the superior sensitivity of molecular diagnostics. Spatial analysis identified significant clustering (Moran’s *I* = 0.330, *p* = 0.0003), with communes-level prevalence ranging from 6.7% to 45.7%. Infection rates correlated positively with humidity and rainfall but negatively with temperature. Phylogenetic analysis confirmed *Cryptosporidium xiaoi* as the sole species circulating, showing 100% genetic similarity to global caprine isolates. Despite *C. xiaoi*’s host adaptation, a GenBank review highlighted six other zoonotic species infecting goats worldwide, underscoring potential cross-species transmission risks. The study emphasizes the need for PCR-based surveillance to assess true prevalence and zoonotic threats, while climatic findings support targeted interventions in high-risk areas.

## 1. Introduction

The apicomplexan protozoans of the genus *Cryptosporidium* are gastrointestinal parasites infecting terrestrial vertebrates (i.e., humans) and aquatic hosts (i.e., fish) worldwide [1,2]. Since the earliest report by Slavin in 1955 on the pathogenic role of *Cryptosporidium meleagridis* causing diarrhea in a turkey farm [3], human cryptosporidiosis remained a rare and opportunistic disease [4,5] until the 1980s, when severe outcomes began to be observed in immunocompromised patients with AIDS [6]. Moreover, a rapid and widespread transmission through contaminated environment and water have been demonstrated in the United States and the United Kingdom [7,8], making it one of the most involved water/food-borne pathogens inducing outbreaks in developed countries [9,10,11]. Basically, when the disease is manifested, infected hosts undergo severe symptoms encompassing diarrhea in newborn individuals (humans and ruminants) to life-threatening complications in immunocompromised patients [12]. However, the infection can be asymptomatic, leading to the presence of hidden reservoirs of *Cryptosporidium* spp. [13,14].

The genus *Cryptosporidium* was first described by Tyzzer more than a century ago, with *C. muris* as the type species [15,16,17]. Currently, species delimitation in *Cryptosporidium* relies on multiple criteria including oocyst morphology, host specificity, genetic characterization, and compliance with the International Code of Zoological Nomenclature (ICZN) [2,18,19]. Recent molecular and biological studies classified the genus as gregarine member of the subclass Cryptogregaria within the class Gregarinomorphea, which was later amended to Cryptogregarinorida within the *Gregarinasina* subclass [20]. This taxonomic complexity is not surprising, as *Cryptosporidium* is part of the large and diverse phylum Apicomplexa, which comprises over 300 genera and more than 6000 species.

Recent studies in genomic and molecular biology highlighted the close relationship between the *Cryptosporidium* species/subspecies and their virulence, pathogenicity, host specificity, and geographical distribution [13,14,21]. However, despite the importance of genetic markers, only a limited number of species were completely resolved among the quested *Cryptosporidium* species [22,23]. Currently, at least 51 valid species of *Cryptosporidium* have been recognized, including recently described species such as *C. sciurinum*, *C. equi*, and *C. mortiferum* [24,25,26], and over 120 genotypes have been identified [27], emphasizing the complexity and diversity within the genus. This genetic variability supports another theory suggesting a low host specificity of *Cryptosporidium* spp., as more than 260 animal species, including humans, have been identified as potential hosts [28], which presents a major challenge for disease control. Domestic and farm animals are frequently infected, making them key players in the transmission of the parasite.

Goats remain important in farming; their frequent exposure to parasites makes them a potential source of infection for humans and other animals. Several *Cryptosporidium* species have been reported from goats worldwide. The most common is *C. xiaoi*, but *C. parvum*, *C. ubiquitum*, and *C. andersoni* are also commonly reported [22]. Less common species like *C. hominis*, *C. bovis*, and *C. ryanae* have also been found in goats [29]. Cryptosporidiosis appears to be a significant threat to the health of neonatal 4–15-day-old goat kids, causing severe diarrhea, dehydration, and high mortality rates if left untreated. The high morbidity and mortality rates linked to this parasitosis often lead to significant economic loss in goat farms, as reported from southern Europe [30].

Understanding the molecular characteristics of *Cryptosporidium* in goats is crucial for implementing effective control strategies to minimize the risk of zoonotic transmission since the truly effective drugs and vaccines against human cryptosporidiosis are absent [31,32]. Along with the high resistance of oocysts in the environment, vigilant surveillance and high hygiene measures are often needed for disease management. To this end, the present study aimed to investigate, genetically, the diversity of *Cryptosporidium* species infecting goats from the Bouira province, Algeria, supported by a comprehensive analysis of all available GenBank data aimed at improving our understanding of the transmission routes, reservoir hosts, and geographical distribution of *Cryptosporidium* parasites.

## 2. Materials and Methods

### 2.1. Sample Collection

From June 2023 to November 2024, a total of 559 fecal samples were collected from 70 farms. Fresh fecal samples were collected from each individual animal on the investigated farms. Samples were collected directly from the rectum using sterile gloves or immediately after natural defecation to avoid environmental contamination. The farms were randomly selected across ten communes in the Bouira province, Algeria. The estimated caprine herd in the Bouira province is estimated to be approximately 3375 goats (data communicated by the “Direction des Services Agricoles ‘DSA’ of Bouira, Algeria”). The collected samples represent approximately 16.6% of the targeted goat population, a sampling ratio considered to be sufficiently representative for studying infectious diseases [33].

All sampled animals appeared to be in good health on the day of collection, with no visible signs of illness. The samples were carefully transported to the laboratory in ice boxes and preserved in 2.5% potassium dichromate at 4 °C until microscopy and molecular analysis.

### 2.2. Light Microscopy Screening

All samples collected underwent microscopic observation using a modified Zielh–Neelsen stain, a negative staining technique for *Cryptosporidium* detection. Briefly, after parasite fixation in 70% methanol for 5 min, fecal smear slides were air-dried and immersed in carbol-fuchsin solution for 1 h. After washing with deionized water, a 2% sulfuric acid solution was used to decolorize the initial stain for 20 s under immersion followed by the washing step as described above. Subsequently, the counterstaining step was performed using a 5% malachite green solution for 5 min followed by the washing step. Finally, slides were air-dried and examined under light microscopy at 1000× magnification (100× objective with immersion oil) for the detection of *Cryptosporidium* oocysts [34].

### 2.3. Molecular Analysis

Individual fecal samples were processed for DNA extraction by homogenizing one gramme of sample in 1 mL of ultrapure distilled water followed by filtration through a mesh to remove debris. An aliquot of 200 µL of the filtrate was then subjected to mechanical lysis using FastPrep-24™ 5G homogenizer under high-speed agitation for 3 cycles of 40 s each in the presence of powder glass. The lysate was subsequently mixed with 200 µL of lysis buffer containing 30% proteinase K and incubated at 56 °C for 24 h. DNA was then extracted using the Thermo Scientific™ KingFisher™ Flex system (London, UK), following the manufacturer’s protocol. The final DNA extract was eluted in 200 µL of distilled water and stored at −20 °C until further analysis.

All samples were screened for *Cryptosporidium* DNA using the genus-specific Real-time qPCR targeting a fragment (∼300 bp) of the small subunit (SSU) 18S rRNA gene as previously described [35]. After the initial screening, all qPCR positive samples were confirmed by nested PCR followed by Sanger sequencing [36]. Briefly, a two-step nested PCR approach was employed to amplify the 18S rRNA gene. In the initial PCR step, a fragment of 763 bp was generated using forward primer 18SiCF2 (5′-GAC ATA TCA TTC AAG TTT CTG ACC-3′) and reverse primer 18SiCR2 (5′-CTG AAG GAG TAA GGA ACA ACC-3′). PCR cycling conditions began with an initial denaturation at 95 °C for 5 min, followed by 45 cycles of denaturation at 95 °C for 30 s, annealing at 58 °C for 30 s, and extension at 72 °C for 30 s. The final extension was performed at 72 °C for 10 min. In the secondary PCR, the target of ∼587 bp fragment was amplified using 1 μL of the primary PCR product and nested forward primer 18SiCF1 (5′-CCT ATC AGC TTT AGA CGG TAG G-3′) and nested reverse primer 18SiCR1 (5′-TCT AAG AAT TTC ACC TCT GAC TG-3′). The cycling conditions for the secondary PCR were identical to those of the primary PCR [35]. All confirmed nested-positive amplicons throughout agarose gel revelation were purified using NucleoFast 96 PCR plates (Macherey-Nagel EURL, Hoerdt, France) prior to the sequencing reaction with the BigDye™ Terminator v3.1 Cycle Sequencing Kit (Applied Biosystems, Foster City, CA, USA). BigDye products were purified using Sephadex G-50 Superfine gel filtration resin and sequenced on an ABI Prism 3130XL sequencer (Applied Biosystems, Foster City, CA, USA).

### 2.4. Phylogenetic Analysis

All DNA amplicons were subjected to visual correction and indel elimination using Chromas software version 2.6.6. The Mafft software version 7 [37] was used to perform multisequence alignment of the DNA sequences against the homologous sequence from the available panel of valid *Cryptosporidium* species identified in caprine hosts, which, to date, includes *C. bovis*, *C. xiaoi*, *C. ryanae*, *C. andersoni*, *C. ubiquitum*, *C. parvum*, and *C. hominis* [22,29]. The multisequence alignment (MSA) was then manually trimmed using BioEdit Software version 7.2 [38]. Phylogenetic analysis was performed using the maximum likelihood method in IQ-TREE software version 2.4.0 [39]. The best phylogenetic model was selected by the Model Finder module and implemented as functionality of the IQ-TREE software and 100 k Bootstraps replications using the following command: (iqutree -s <input.fast> --auto -m TEST -bb 100,000). For a more informative phylogenetic tree, a table of all sequence records of the quested species (i.e., *C. bovis*, *C. xiaoi*, *C. ryanae*, *C. andersoni*, *C. ubiquitum*, *C. parvum*, and *C. hominis*) from all hosts including caprine was retrieved and used to shed light on geographical and host diversity. Finally, the newish phylogeny file was displayed and edited on iTOL software version 7.1 [40].

### 2.5. Data Collection and Preprocessing

Molecular and microscopy screening data, the 2024 weather and climate data (i.e., average humidity, temperature, and rainfall) for the Bouira province retrieved from (https://weatherandclimate.com/algeria/bouira#google_vignette, accessed on 1 November 2024) [41], and the commune-level geographical boundaries of Algeria retrieved from SimpleMaps web server (https://simplemaps.com/gis/country/dz#all, accessed on 1 November 2024) were used for the epidemiological analysis. Prevalence was calculated for communes (*n* = 10) and breeding units (*n* = 70), with Wilson score intervals providing a 95% confidence interval. McNemar’s test was used for paired comparison between molecular and microscopy screening. To assess whether the observed prevalence was significantly different from a theoretical baseline of 5%, a two-tailed z-test for one proportion was applied for each group (H₀: *p* = 0.05). The corresponding *p*-value was computed, and statistical significance was defined as *p* < 0.05. Climate correlations used Pearson’s r with Fisher-transformed CIs, while spatial autocorrelation was assessed via Moran’s I (Queen contiguity). The Folim library version (0.19.5) was used to create interactive leaflet maps for prevalence gradients and climate overlay visualizations. Seaborn (v0.13) regression plots of climate–prevalence relationships were also performed. All analyses were conducted in Python (3.8) using pandas, stats models, and geospatial libraries, with automated reporting ensuring reproducibility.

## 3. Results

### 3.1. Cryptosporidium Screening

Morphological detection of *Cryptosporidium* using microscopy screening yielded the identification of 34 positive samples, which corresponds to a prevalence of 6.1%: 95% CI (4.4–8.4) (Figure S1, Table 1). However, molecular screening revealed a total of 76 positive samples (13.6% (95% CI: 11.0–16.7)) and confirmed all microscopy-positive samples, making qPCR detection significantly more sensitive than microscopy (McNemar’s Test, *p*-value < 0.00001). Overall, there was a significant variation in *Cryptosporidium* prevalence across communes and breeding sites (Table 1). Commune-level prevalence ranged from 6.7% (Bouira) to 45.7% (El Adjiba), with infection rates in positive breeding sites varying widely (11.1–58.3%). Among breeding sites with at least one infected animal, prevalence reached 41.5% (95% CI: 34.2–49.3), compared to 13.6% (95% CI: 11.0–16.7) in the overall population (N = 559). Statistical significance (*p* < 0.05) was observed in multiple breeding sites, particularly in high-prevalence communes like El Adjiba and Bechloul.

**Figure 1:**
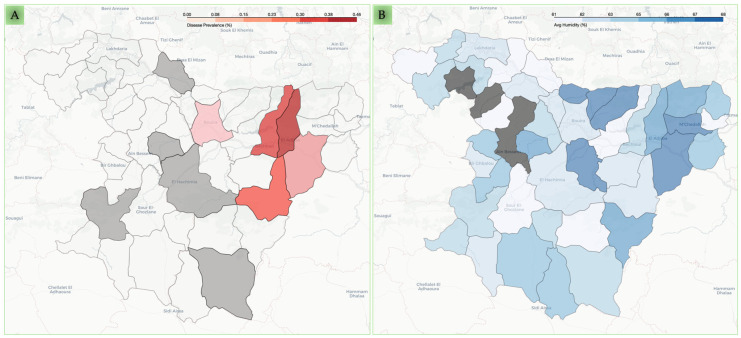
Spatial distribution of caprine cryptosporidiosis prevalence and environmental humidity across Bouira communes. Panel (**A**) displays the molecular prevalence of caprine cryptosporidiosis in the surveyed communes. The intensity of the red shading indicates higher prevalence, while grey-shaded communes represent areas with no reported cases from the investigated caprine breeding and white-shaded communes represent the remaining communes with missing data. Panel (**B**) illustrates the mean annual humidity (%) across the same communes. The intensity of the blue shading indicates higher humidity levels. Grey represents communes for which climate data were unavailable.

In addition, the prevalence of *Cryptosporidium* infection seems to be geographically related, as demonstrated by the Moran’s I test for spatial autocorrelation: 0.330 (*p* = 0.0003). Pearson’s positive correlation with *Cryptosporidium* infection was observed for the average humidity (r = 0.308) and the rainfall rate (r = 0.289) in contrast to the average temperature (r = −0.259) for the endemic communes, as shown in Figure 1A,B and Figure S1, and File S1.

### 3.2. Genetic Study

**Figure 2:**
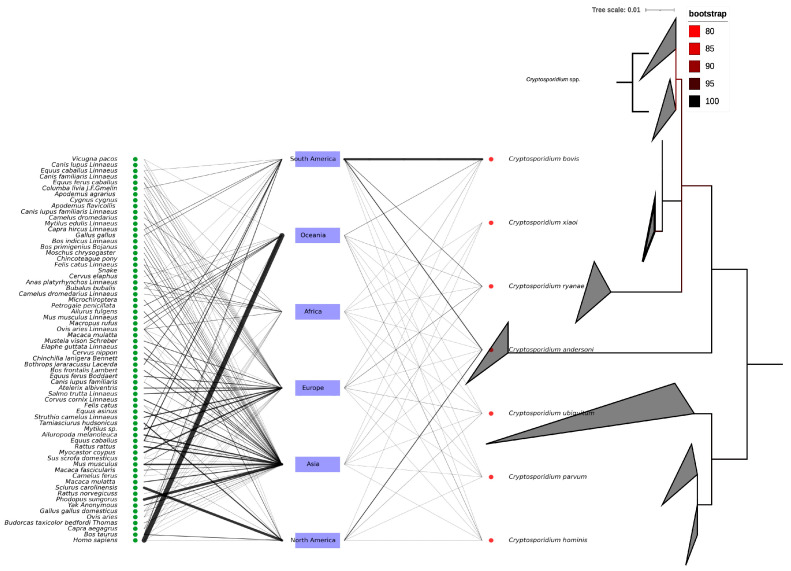
Evolutionary relationship of caprine-associated *Cryptosporidium* spp. The right cladogram represents the Maximum Likelihood phylogenetic tree of *Cryptosporidium* sequences (*n* = 179) from goat hosts. The tree was inferred using IQ-TREE under the TPM2u + F + I substitution model, with ultrafast bootstrap support values (100,000 replicates). The tree is midpoint-rooted for clarity. Branch lengths represent the number of substitutions per site. Sequence clades are labeled with their species name. The scale bar indicates the number of substitutions per site. The bipartite network of continents, hosts, and *Cryptosporidium* species infecting caprine.

The 18S rRNA PCR yielded the amplification and sequencing of 61 out of the 76 qPCR positive samples for *Cryptosporidium* DNA. All DNA sequences were deposited in the GenBank database under the following accession numbers: PV569647-PV569707. All DNA sequences were identical and showed 100% identity, with *C. xiaoi* previously isolated from a caprine host in Poland (KY055406), from slaughterhouse wastewater in Iran (KT175422), and from diarrheic caprine feces in South Korea (OQ928535). Likewise, the ML phylogeny confirmed the species identification of the isolates as *C. xiaoi*, as shown in Figure 1. In addition, the exploratory analysis of the sequence records from the GenBank database highlighted the occurrence of at least seven *Cryptosporidium* species (i.e., *C. bovis*, *C. xiaoi*, *C. ryanae*, *C. andersoni*, *C. ubiquitum*, *C. parvum*, and *C. hominis*) within caprine hosts worldwide. These species also share at least 66 vertebrate hosts other than caprine, as shown in Figure 2.

## 4. Discussion

The present study identified *Cryptosporidium xiaoi* as the sole species infecting goats in domestic farms in Bouira, Algeria, with a prevalence rate consistent with previous reports in central Algeria (17.02%) [42] but higher than earlier findings from other regions (5.19–8.7%) [43].

Globally, *Cryptosporidium* prevalence in goats varies widely (0–100%) [44], influenced by factors such as animal age, diagnostic methods, husbandry practices, and climate. High infection rates have been reported in industrialized nations (7.1–93.0%) [45,46] and developing countries (2.9–72.5%) [47,48,49,50,51,52], with extreme cases like 100% in Italy [53]. The dominance of *C. xiaoi* infection in goats is not new and has been reported from several parts of the world such as Africa (Algeria, Egypt, Tanzania) [43,48,54], Asia (Bangladesh, China) [49,50], and Europe (France, Greece, Norway) [44,55,56], suggesting its adaptability to small ruminants.

Goats, often kept in small-scale or backyard farms, may act as hidden reservoirs for *Cryptosporidium*, posing zoonotic risks. Our study revealed a low sensitivity of microscopy method in detecting *Cryptosporidium* infection compared to molecular diagnostics. Microscopy’s limitations are due to small oocyst size, morphological similarities to yeast, and intermittent shedding, reducing accuracy [57,58], whereas PCR shows the ability to detect microscopy-negative cases [59,60]. This is particularly important in caprine populations, where *Cryptosporidium* species can have zoonotic potential. Inadequate detection may mask the true extent of infection and the associated public health risks. Therefore, our findings support the use of combined diagnostic approaches for accurate prevalence assessment and public health risk management.

Although *C. xiaoi* is generally considered a host-adapted species with limited zoonotic potential, our GenBank exploratory analysis revealed that goats worldwide may host at least six other species, including *C. parvum*, *C. hominis*, *C. bovis*, *C. ryanae*, *C. andersoni*, and *C. ubiquitum*, many of which can infect a wide range of vertebrate hosts [22,29]. This co-occurrence raises the possibility of cross-species transmission and even interspecific recombination, especially in mixed infections [22]. In this light, the uniformity of *C. xiaoi* in our samples may reflect either a true clonal expansion, like the global spread of *C. hominis* IbA10G2 [61], or a limitation of single-locus genotyping herein deployed [22]. As highlighted by Feng et al. (2018), multi-locus sequence typing (MLST) and whole-genome sequencing (WGS) approaches are essential to reveal cryptic diversity, clarify host specificity and adaptation mechanisms, and provide a more comprehensive understanding of the evolutionary dynamics of *Cryptosporidium* in goats [22].

Despite the advances in molecular tools to detect and identify *Cryptosporidium* infection from human and animal species, the caprine cryptosporidiosis remains limited to only seven *Cryptosporidium* species based on genetic data. The low *Cryptosporidium* diversity may be due to the limited investigation of caprine hosts. For example, previous studies reported the occurrence of other zoonotic species (i.e., *C. parvum*, *C. hominis*) in Algeria, but caprine-specific sequences remain underrepresented with a rate of 0.17% of global *Cryptosporidium* records. Regional variations in species distribution are linked to farming practices and climate [62]. For instance, *C. parvum* and *C. ubiquitum* are more common in young goats and carry zoonotic potential [62], as seen in South Korea, where diarrheic cases showed a sympatric occurrence of both species [63]. Outbreaks are often tied to intensive farming and humid conditions, exacerbating neonatal diarrhea and weight loss due to massive *Cryptosporidium* propagation [55]. Previous observations emphasized the influence of climatic variables, particularly humidity, precipitation, and temperature, on the incidence and spread of cryptosporidiosis [64]. The spatial analysis in the present study showed a cluster-related distribution of *Cryptosporidium* over the investigated communes (Moran’s I = 0.33), correlating positively with humidity (*r* = 0.308) and rainfall (*r* = 0.289) but negatively with temperature (*r* = −0.259). Similar trends were noted in Australia [65], Scotland [66], and Canada [67], where moisture facilitates oocyst survival. The inverse temperature correlation may reflect local behavioral or environmental factors, such as reduced communal water source usage in heat. Geostatistical tools, as applied here, can pinpoint high-risk zones, guiding targeted interventions during peak transmission seasons.

## 5. Conclusions

In conclusion, *C. xiaoi* predominance in Algerian goats reflects global trends, but underreporting may obscure true species diversity. PCR-based diagnostics are critical for accurate surveillance, especially given goats’ role as potential zoonotic reservoirs. Climatic factors significantly influence transmission, necessitating seasonally adaptive control measures. Future research should prioritize genomic tools to elucidate strain diversity and zoonotic risks, informing One Health strategies to mitigate cryptosporidiosis in livestock and humans.

## Figures and Tables

**Table 1:** Detailed results of molecular screening of *Cryptosporidium* DNA in caprine breeding from Bouira.

Commune	Breeding Id		Nb. of Samples	Molecular Prevalence	Microscopy Prevalence	*p*-Value
Ahnif Total tested: 52 Prevalence: 11.5 95% CI: (5.5–23.0) *p*-value: 0.139	AhB1		9	11.1 [1.99–43.50]	11.1 [1.99–43.50]	0.5596
	AhB2		7	28.6 [8.22–64.11]	14.3 [2.57–51.31]	0.1674
	AhB6		6	50 [18.76–81.24]	16.7 [3.01–56.35]	0.0275
Bechloul Total tested: 66 Prevalence: 33.3 95% CI: (23.2–45.3) *p*-value: <0.00001	BeB1		10	40 [16.82–68.73]	10 [1.79–40.41]	0.0239
	BeB2		9	33.3 [12.06–64.58]	33.3 [12.06–64.58]	0.0714
	BeB3		8	25 [7.15–59.07]	25 [7.15–59.071]	0.1914
	BeB4		11	18.2 [5.14–47.70]	ND	0.257
	BeB5		7	42.9 [15.82–74.95]	28.6 [8.22–64.11]	0.043
	BeB6		12	50 [25.38–74.62]	41.7 [19.33–68.049]	0.0018
	BeB7		9	22.2 [6.32–54.74]	11.1 [1.99–43.50]	0.214
Bouira Total tested: 45 Prevalence: 6.7 95% CI: (2.3–17.8) *p*-value: 0.654	BoB1		6	50 [18.76–81.24]	50 [18.76–81.24]	0.0275
El Adjiba Total tested: 70 Prevalence: 45.7 95% CI: (34.6–57.3) *p*-value: <0.00001	AdB1		10	30 [10.78–60.32]	ND	0.0845
	AdB2		11	54.5 [28.01–78.73]	9.1 [1.62–37.74]	0.001
	AdB3		9	55.6 [26.67–81.12]	33.3 [12.06–64.58]	0.0023
	AdB4		11	54.5 [28.01–78.73]	18.2 [5.14–47.70]	0.001
	AdB5		10	50 [23.66–76.34]	10 [1.79–40.42]	0.0044
	AdB6		12	58.3 [31.95–80.67]	8.3 [1.49–35.39]	0.0002
Ouled Rached Total tested: 53 Prevalence: 24.5 95% CI: (14.9–37.6) *p*-value: 0.0009	OuB5		9	44.4 [18.88–73.33]	33.3 [12.06–64.58]	0.0172
	OuB6		7	57.1 [25.05–84.18]	14.3 [2.57–51.31]	0.0053
	OuB7		10	50 [23.66–76.34]	20 [5.67–50.98]	0.0044
Prevalence in infected breeding	qPCR	N = 20	183	41.5 [34.2–49.3]	-	χ^2^ = 212.0
	Microscopy	N = 18	162	-	20 [15.43–27.89]	
Global prevalence	N = 70		559	13.6 [11.00–16.69]	6.1 [4.38–8.38]	χ^2^ = 29.9

ND: Not detected. All comparative statistics were performed on the molecular results.

## Data Availability

All data used are shared in the core text or Supplemntary Data.

## Supplementary Materials

The following supporting information can be downloaded at: https://www.mdpi.com/article/10.3390/pathogens14060597/s1, Figure S1: Relationship between climate variables (humidity, temperature, rainfall) and caprine *Cryptosporidium* prevalence in Bouira province, Algeria; File S1: Summary statistics of *Cryptosporidium* prevalence in Bouira province, Algeria.
