# Supplementary figures and images for: Molecular and Epidemiological Investigation of Cryptosporidium Infection in Goat Population from Bouira Province, Algeria
Source: Pathogens. 2025 Jun 18;14(6):597. doi: 10.3390/pathogens14060597 (PMC12196339; doi:10.3390/pathogens14060597)

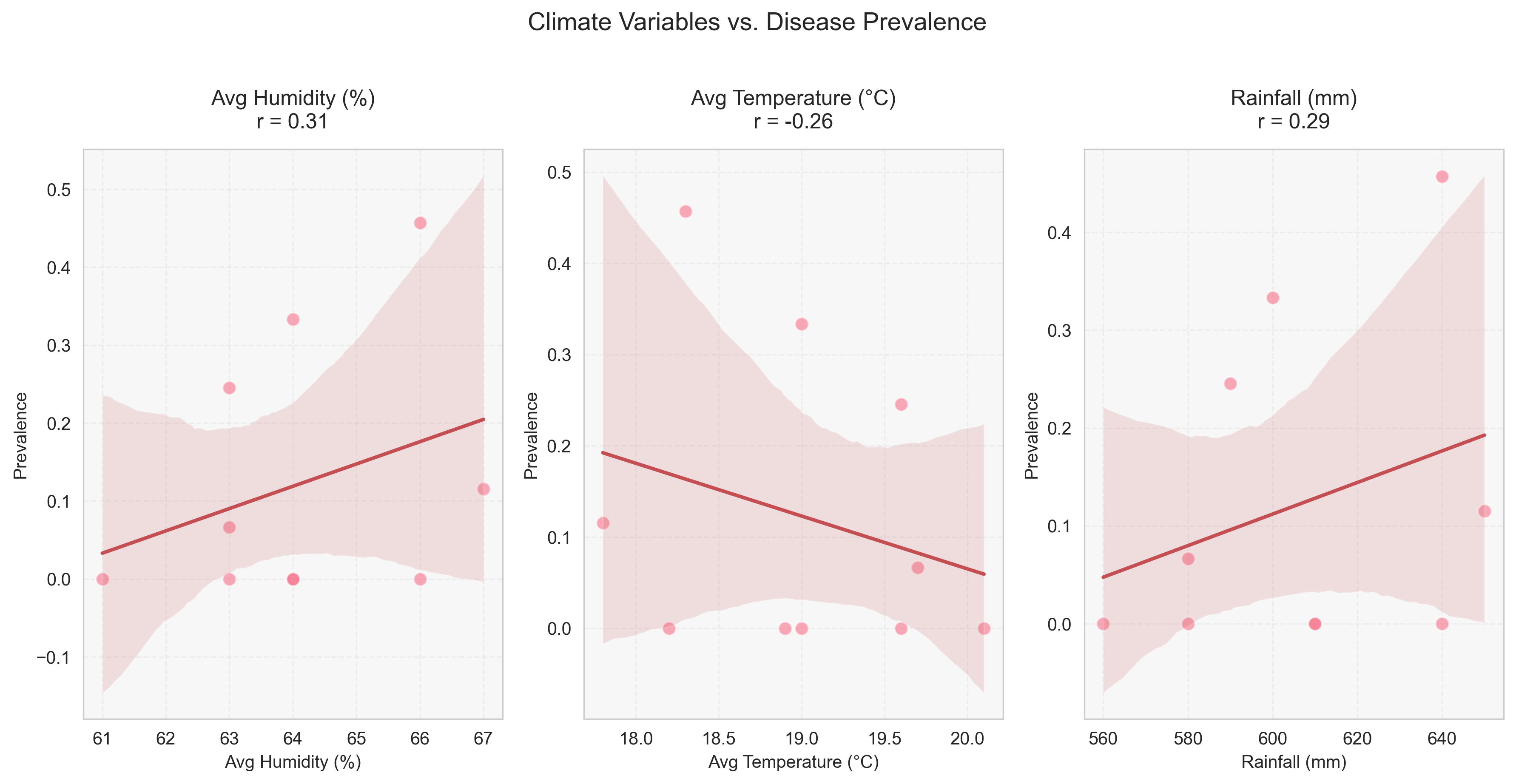

Supplement: Supplementary file 1 [file pathogens-14-00597-s001.zip › Figure S1.png]
